# Supplementary material for: A beta-Poisson model for infectious disease transmission
Source: PLoS Comput Biol. 2024 Feb 8;20(2):e1011856. doi: 10.1371/journal.pcbi.1011856 (PMC10903957; doi:10.1371/journal.pcbi.1011856)
Supplement: S1 Appendix — Fig A. Log-likelihood curves of the beta-Poisson model parameters by dataset. Table A. Maximum likelihood estimates of negative binomial model parameters by dataset. Table B. Maximum likelihood estimates of zero-inflated Poisson model parameters by dataset. (PDF) [file pcbi.1011856.s001.pdf]

Appendix to *A beta-Poisson model for infectious disease transmission*

Joe Hilton[1], Ian Hall[2*]

1 School of Life Sciences and Zeeman Institute (SBIDER), University of Warwick, Coventry, UK
2 Department of Mathematics and School of Health Sciences, University of Manchester, Manchester, UK

* ian.hall@manchester.ac.uk

The model description in Section 1 of this Appendix and the log-likelihood formula in Section 2 are sufficient to reproduce the results presented in the main text. To make our account of the beta-Poisson model more complete, in the rest of this Appendix we outline moment calculations (Section 3), the probability generating function (Section 4), the branching process extinction probability (Section 5), the minor outbreak size distribution (Section 6), the distribution of attack rates in a major outbreak (Section 7), and likelihood calculations using outbreak size data (Section 8).

1 Model description

The beta-Poisson model describes the person-to-person spread of a pathogen in a population where contact behaviour is homogeneous but transmission behaviour varies from person to person. We assume that during their infectious period an infectious individual makes a Poisson-distributed number of contacts with mean N. For each case a transmission probability p is chosen from a beta distribution with parameters (α_1, α_2), and each of their contacts independently results in a new infection with probability p. As was outlined in the Methods section of the main text, the resulting total number of infections generated during an individual's infectious period conditioned on p is Poisson distributed with mean pN. With this in mind, we can integrate with respect to p to obtain the probability density function of $x \in \mathbb{N} \cup \{0\}$, the number of secondary cases generated by a single case in the beta-Poisson epidemic model:

$$\begin{aligned} P(x : \alpha_1, \alpha_2, N) &= \int_0^1 \frac{p^{\alpha_1 - 1}(1-p)^{\alpha_2 - 1}}{B(\alpha_1, \alpha_2)} \frac{(pN)^x e^{-pN}}{\Gamma(x+1)} \mathrm{d}p \\ &= \frac{N^x}{B(\alpha_1, \alpha_2)\Gamma(x+1)} \int_0^1 e^{-pN} p^{x+\alpha_1-1}(1-p)^{\alpha_2-1} \mathrm{d}p \\ &= \frac{N^x}{\Gamma(x+1)} \frac{\Gamma(x+\alpha_1)\Gamma(\alpha_1+\alpha_2)}{\Gamma(\alpha_1)\Gamma(x+\alpha_1+\alpha_2)} M(x+\alpha_1, x+\alpha_1+\alpha_2, -N), \end{aligned} \tag{1}$$

where $M(a, b, x)$ is the confluent hypergeometric function. We will use several properties of this function when required but will not discuss its properties in detail. For more information see, for example, Abramowitz and Stegun [1].

We can calculate the mean λ of the beta-Poisson distribution using the Law of Total Expectation, where X is a beta-Poisson distributed random variable which is

conditional on the underlying beta-distributed random variable $P \sim \text{Beta}(\alpha_1, \alpha_2)$:

$$\begin{aligned}\lambda &= \mathbb{E}[\mathbb{E}[X|P]] \\ &= \mathbb{E}[N \cdot P] \\ &= N\mathbb{E}[P] \\ &= N\frac{\alpha_1}{\alpha_1 + \alpha_2}.\end{aligned} \tag{2}$$

From this is is immediately clear that $\lambda \leq N$. The notation λ is chosen by analogy with the standard notation for the mean of the (unmixed) Poisson distribution, and in our epidemiological application λ is the basic or effective reproductive ratio (depending on context). The beta distribution parameters α_1 and α_2 lack an intuitive interpretation in terms of transmission behaviour, but by making the substitution $\Phi = \frac{\alpha_1+\alpha_2}{N}$, so that $\alpha_1 = \Phi N$ and $\alpha_2 = \Phi(N - \lambda)$, we can express the distribution in terms of the mean λ, Φ, and N:

$$P(x; \lambda, \Phi, N) = \frac{N^x}{\Gamma(x+1)} \frac{\Gamma(x + \Phi\lambda)\Gamma(\Phi N)}{\Gamma(\Phi\lambda)\Gamma(x + \Phi N)} M(x + \Phi\lambda, x + \Phi N, -N). \tag{3}$$

When its first two arguments are equal, the confluent hypergeometric function is given by the exponential of its third argument, and so the beta-Poisson distribution reduces to the (unmixed) Poisson distribution when $\lambda = N$.

In the limit $N \to \infty$, the beta-Poisson distribution is equivalent to the negative binomial distribution with parameters λ and $\theta = \Phi^{-1}$. To see this, we first note that

$$\lim_{n\to\infty} n\text{Beta}(k, n) = \text{Gamma}(k, 1)$$

and that

$$\text{Gamma}(\alpha, 1) = \beta^{-1}\text{Gamma}(\alpha, \beta).$$

From the definition of the beta-Poisson distribution, a beta-Poisson random variable is drawn from a Poisson distribution with mean Np where $p \sim \text{Beta}(\lambda\Phi, (N - \lambda)\Phi)$. From the identities stated above it follows that

$$\begin{aligned}\lim_{N\to\infty} N\text{Beta}(\lambda\Phi, (N-\lambda)\Phi) &= \Phi^{-1}\text{Gamma}(\lambda\Phi, 1) + \lambda\Phi \lim_{N\to\infty} \text{Beta}(\lambda\Phi, (N-\lambda)\Phi) \\ &= \text{Gamma}(\lambda\Phi, \Phi^{-1}) + \lambda\Phi \lim_{N\to\infty} \text{Beta}(\lambda\Phi, (N-\lambda)\Phi).\end{aligned} \tag{4}$$

It follows that the beta-Poisson distribution can be expressed as the sum of a Gamma-Poisson mixture with Gamma parameters $\frac{\lambda}{\theta}$ and $\theta = \Phi^{-1}$ and a beta-Poisson mixture with parameters $\lambda\Phi$ and $(N - \lambda)\Phi$, with the latter term scaled by a factor of $\lambda\Phi$. This Poisson-beta mixture is slightly different to the beta-Poisson model we are considering in this study, since our model applies a scaling of N to the beta-distributed random variable before feeding it into the Poisson distribution. The rth moment of a beta distribution with parameters (α_1, α_2) is given by the product

$$\prod_{j=0}^{r-1} \frac{(\alpha_1 + j)}{\alpha_1 + \alpha_2 + j},$$

and so in the limit $N \to \infty$, all of the moments of the distribution $\text{Beta}(\lambda\Phi, (N - \lambda)\Phi)$ will tend to zero. In Section 3 of this Appendix we show that because the rth moment of a mixed Poisson distribution is given by a weighted sum of the first r moments of its mixing distribution [2], the moments of the beta-Poisson mixture will also tend to zero as N tends to infinity. Thus, as $N \to \infty$, this beta-Poisson mixture will tend to a point

mass at zero. This leaves us with only the Gamma-Poisson mixture term of our sum, which is precisely the negative binomial distribution with parameters (λ, θ).

Using the same random variables as in Equation2, we can apply the Law of Total Variance to derive the variance of the beta-Poisson distribution. Rearranging the definition of Φ to get $\alpha_1 + \alpha_2 = N\Phi$, we obtain:

$$\begin{aligned}\sigma^2 &= \mathbb{E}[\mathrm{Var}(X|P)] + \mathrm{Var}(\mathbb{E}[X|P]) \\ &= \mathbb{E}[N \cdot P] + \mathrm{Var}(N \cdot P) \\ &= \lambda + N^2 \mathrm{Var}(P) \\ &= \lambda + N^2 \frac{\alpha_1 \alpha_2}{(\alpha_1 + \alpha_2)^2 (\alpha_1 + \alpha_2 + 1)} \\ &= \lambda + N^2 \frac{\lambda\Phi \cdot (N - \lambda)\Phi}{(N\Phi)^2 (N\Phi + 1)} \\ &= \lambda + \frac{\lambda(N - \lambda)}{N\Phi + 1} \\ &= \lambda(1 + \frac{N - \lambda}{N\Phi + 1}).\end{aligned} \tag{5}$$

The variance-to-mean ratio ϵ is given by

$$\begin{aligned}\epsilon &= 1 + \frac{N - \lambda}{N\Phi + 1} \\ &= 1 + \frac{1 - \frac{\lambda}{N}}{\Phi + \frac{1}{N}}.\end{aligned} \tag{6}$$

In the limit $N \to \infty$, $\epsilon = 1 + \frac{1}{\Phi}$, in keeping with our interpretation of Φ as the reciprocal of the overdispersion parameter θ from the negative binomial distribution. When $\lambda = N$ the variance-to-mean ratio is 1, in keeping with our earlier statement that this is the (unmixed) Poisson distribution.

We can express the standard beta distribution parameters as follows:

$$\begin{aligned}\alpha_1 &= \lambda\Phi \\ &= \lambda(\frac{1 - \frac{\lambda}{N}}{\epsilon - 1} - \frac{1}{N}) \\ &= \frac{\lambda}{N}(\frac{N - \lambda}{\epsilon - 1} - 1), \\ \alpha_2 &= (N - \lambda)\Phi \\ &= \frac{N - \lambda}{\lambda}\frac{\lambda}{N}(\frac{N - \lambda}{\epsilon - 1} - 1) \\ &= (1 - \frac{\lambda}{N})(\frac{N - \lambda}{\epsilon - 1} - 1).\end{aligned} \tag{7}$$

Since both these quantities are strictly positive and $N \geq \lambda$, we see that $N - \lambda \geq \epsilon - 1$ and $\epsilon \geq 1$. The condition that $\epsilon \geq 1$ means the distribution is overdispersed relative to the Poisson parameter except when $\lambda = N$, i.e. $\alpha_2 = 0$. In this scenario the beta mean is 1 and so all of the binomial transmission trials are successful, meaning our offspring distribution is precisely the Poisson distribution with mean N. We also have an upper bound on the variance, $\sigma^2 \leq (1 + N - \lambda)\lambda$.

2 Likelihood calculations using secondary case data

In this section we outline the estimation of the beta-Poisson model's parameters given a set of secondary case data $\mathbf{x} = (x_1, ..., x_K)$, where x_i is the number of infections

generated by the ith case in the outbreak. The log-likelihood of parameters (λ, Φ, N) given $\mathbf{x}$ is given by

$$\begin{aligned}\log \mathcal{L} &= \sum_{i=1}^{K} \log P(x_i; \lambda, \phi, N) \\ &= \sum_{i=1}^{K} \log \left(\frac{N_i^x}{\Gamma(x_i+1)} \frac{\Gamma(x_i+\Phi\lambda)\Gamma(\Phi N)}{\Gamma(\Phi\lambda)\Gamma(x_i+\Phi N)} M(x_i+\Phi\lambda, x_i+\Phi N, -N) \right) \\ &= \sum_{i=1}^{K} x_i \log N - \log\Gamma(x_i+1) + \log\Gamma(x_i+\Phi\lambda) + \log\Gamma(\Phi N) - \\ &\qquad \log\Gamma(\Phi\lambda) - \log\Gamma(x_i+\Phi N) + \log M(x_i+\Phi\lambda, x_i+\Phi N, -N).\end{aligned} \tag{8}$$

The confluent hypergeometric function is differentiable in each of its arguments so long as all are positive, and so this log likelihood expression can be differentiated through application of the chain rule [3]. However, the derivatives of the confluent hypergeometric function with respect to its first two arguments lack easily derivable closed form solutions suitable for finding maximum likelihood estimates analytically. A general result from the theory of branching processes states that the MLE of the mean of a Galton-Watson process given an observation of the number of particles $Z_0, ..., Z_T$ in successive generations $0, ..., T$ of the process (where Z_0 represents the initial population) is equal to $(Z_1 + ... + Z_T)/(Z_0 + ... + Z_{T-1})$, i.e. the total number of particles generated in the process divided by the number of particles which generated them [4]. In the context of secondary case data, the numerator of this fraction is given by the sum of all the secondary case numbers while the denominator is given by the number of datapoints, since this is the total number of cases who are responsible for the other cases. This fraction is precisely the sample mean of the secondary case data, and so the MLE of the reproductive ratio λ is given by the sample mean, $\bar{x}$. This reduces the calculation of an MLE to a two-dimensional problem which can be solved numerically as outlined in the Methods section of the paper.

3 Moment calculations

The moments of the beta-Poisson distribution are relatively easy to calculate. For a mixed Poisson distribution with rate Y (where Y is some random variable), the rth moment $\mathbb{E}[X^r]$ is given by

$$\mathbb{E}[X^r] = \sum_{j=1}^{r} S(r, j)\mathbb{E}[Y^r], \tag{9}$$

where $S(r, j)$ denotes the Stirling numbers of the second kind [2]. In our model, $Y = Np$, so $\mathbf{E}[Y^r] = N^r \mathbb{E}[p^r]$. Using the beta function identity

$$\frac{B(\alpha_1+1, \alpha_2)}{B(\alpha_1, \alpha_2)} = \frac{\alpha_1}{\alpha_1+\alpha_2}, \tag{10}$$

we find that

$$\begin{aligned}\mathbb{E}[p^r] &= \int_0^1 \frac{p^{\alpha_1+r-1}(1-p)^{\alpha_2-1}}{B(\alpha_1,\alpha_2}\mathrm{d}p \\ &= \frac{B(\alpha_1+r,\alpha_2)}{B(\alpha_1,\alpha_2)} \\ &= \frac{\alpha_1^{(r)}}{(\alpha_1+\alpha_2)^{(r)}} \\ &= \frac{(\lambda\Phi)^{(r)}}{(N\Phi)^{(r)}},\end{aligned} \tag{11}$$

where we have applied Equation 10 repeatedly to write the ratio of beta functions in terms of the Pochhamer function defined by

$$x^{(r)} = \prod_{i=0}^{r-1}(x+i). \tag{12}$$

It follows that the rth moment of the beta-Poisson distribution is given by the formula

$$\mathbb{E}[x^r] = \sum_{j=1}^{r} S(r,j)N^r\frac{(\lambda\Phi)^{(r)}}{(N\Phi)^{(r)}} \tag{13}$$

Using the fact that $S(2,1)=S(2,2)=1$ (see, for example, Abramowitz and Stegun [1]), for $r=2$ we find

$$\begin{aligned}\mathbb{E}[x^2] &= N\frac{\lambda\Phi}{N\Phi} + N^2\frac{\lambda\Phi(\lambda\Phi+1)}{N\Phi(N\Phi+1)} \\ &= \lambda(1+\frac{N(\lambda\Phi+1)}{N\Phi+1}),\end{aligned} \tag{14}$$

and so

$$\begin{aligned}\sigma^2 &= \lambda(1+\frac{N(\lambda\Phi+1)}{N\Phi+1}) - \lambda^2 \\ &= \lambda(1+\frac{N(\lambda\Phi+1)}{N\Phi+1} - \lambda) \\ &= \lambda(1+\frac{N(\lambda\Phi+1)-\lambda(N\Phi+1)}{N\Phi+1}) \\ &= \lambda(1+\frac{N-\lambda}{N\Phi+1}),\end{aligned} \tag{15}$$

in agreement with the expression we found through direct calculation in the Section 1 of this Appendix.

Alongside the mean and variance, another moment which may be of interest in the context of outbreak modelling is kurtosis. This can be interpreted as measuring a distribution's "tailedness", which is of interest since superspreading events by definition belong to the tail of the offspring distribution. The kurtosis is given by

$$\mathrm{Kurt}[X] = \frac{\mathbb{E}[(x-\mathbb{E}[x])^4]}{\sigma^4}. \tag{16}$$

Using Equation 9, the calculation of kurtosis is as follows (we multiply by σ^4 for notational convenience, since nothing particularly cancels out):

$$\begin{aligned}\sigma^4\mathrm{Kurt} &= \mathbb{E}[x^4 - 4\lambda x^3 + 6\lambda^2x^2 - 4\lambda^3x + \lambda^4] \\ &= \mathbb{E}[x^4] - 4\lambda\mathbb{E}[x^3] + 6\lambda^2\mathbb{E}[x^2] - 4\lambda^3\mathbb{E}[x] + \lambda^4.\end{aligned} \tag{17}$$

Using Equation 9 and substituting in the required Stirling numbers, we get the following four equations for the moments of x:

$$\begin{aligned}\mathbb{E}[x] &= N\mathbb{E}[p]\\ \mathbb{E}[x^2] &= N\mathbb{E}[p] + N^2\mathbb{E}[p^2]\\ \mathbb{E}[x^3] &= N\mathbb{E}[p] + 3N^2\mathbb{E}[p^2] + N^3\mathbb{E}[p^3]\\ \mathbb{E}[x^4] &= N\mathbb{E}[p] + 7N^2\mathbb{E}[p^2] + 6N^3\mathbb{E}[p^3] + N^4\mathbb{E}[p^4].\end{aligned} \tag{18}$$

Substituting these expressions into Equation 17, we get

$$\begin{aligned}\sigma^4\mathrm{Kurt} = &\quad N\mathbb{E}[p] + 7N^2\mathbb{E}[p^2] + 6N^3\mathbb{E}[p^3] + N^4\mathbb{E}[p^4]\\ &- 4N\lambda\mathbb{E}[p] - 12N^2\lambda\mathbb{E}[p^2] - 4N^3\lambda\mathbb{E}[p^3]\\ &+6N\lambda^2\mathbb{E}[p] + 6N^2\lambda^2\mathbb{E}[p^2]\\ &-4N\lambda^3\mathbb{E}[p]\\ &+\lambda^4\\ = &\quad N^4\mathbb{E}[p^4] + (6-4\lambda)N^3\mathbb{E}[p^3] + (6\lambda^2 - 12\lambda + 7)N^2\mathbb{E}[p^2]\\ &-(4\lambda^3 - 6\lambda^2 + 4\lambda - 1)N\mathbb{E}[p] + \lambda^4.\end{aligned} \tag{19}$$

Since $N\mathbb{E}[p] = \lambda$, the last two terms add together to give

$$\begin{aligned}-(4\lambda^3 - 6\lambda^2 + 4\lambda - 1)\lambda + \lambda^4 &= -\lambda(3\lambda^3 - 6\lambda^2 + 4\lambda - 1)\\ &= -\lambda(\lambda - 1)(3\lambda^2 - 3\lambda + 1).\end{aligned} \tag{20}$$

Substituting this and Equation 11 into Equation 19, we get

$$\begin{aligned}\sigma^4\mathrm{Kurt} = &\quad N^4\frac{\lambda\Phi^{(4)}}{N\Phi^{(4)}} + (6-4\lambda)N^3\frac{\lambda\Phi^{(3)}}{N\Phi^{(3)}} + (6\lambda^2 - 12\lambda + 7)N^2\frac{\lambda\Phi^{(2)}}{N\Phi^{(2)}}\\ &-\lambda(\lambda - 1)(3\lambda^2 - 3\lambda + 1).\end{aligned} \tag{21}$$

We leave the Pochhammer symbols as is, since the formula is unlikely to clean up any more than this. Since σ^2 is linear in λ, it follows that the kurtosis will end up being quadratic in λ.

4 Probability generating function

The beta-Poisson distribution's probability generating function (PGF) is necessary for calculating the extinction probability and outbreak size distribution.

The PGF of a mixed Poisson distribution with mixing distribution pdf $g(\lambda)$ is given by [2]

$$G(s) = \int_0^\infty e^{\lambda(s-1)} g(\lambda)\mathrm{d}\lambda. \tag{22}$$

Thus, the PGF of a beta-Poisson distributed random variable X is

$$\begin{aligned} G_X(s) &= \int_0^1 e^{pN(s-1)} \frac{p^{\alpha_1-1}(1-p)^{\alpha_2-1}}{\mathbf{B}(\alpha_1,\alpha_2)} \mathrm{d}p \\ &= \frac{1}{\mathbf{B}(\alpha_1,\alpha_2)} \int_0^1 e^{pN(s-1)} p^{\alpha_1-1}(1-p)^{\alpha_2-1} \mathrm{d}p \\ &= \frac{1}{\mathbf{B}(\alpha_1,\alpha_2)} \frac{\Gamma(\alpha_1)\Gamma(\alpha_2)}{\Gamma(\alpha_1+\alpha_2)} M(\alpha_1, \alpha_1+\alpha_2, N(s-1)) \\ &= M(\alpha_1, \alpha_1+\alpha_2, N(s-1)) \\ &= M(\lambda\Phi, N\Phi, N(s-1)). \end{aligned} \tag{23}$$

The PGF of the random variable Z, the total cases generated by K cases, is then

$$G_Z(s) = (M(\lambda\Phi, N\Phi, N(s-1)))^K. \tag{24}$$

5 Extinction probability

Extinction occurs with probability one for branching process epidemics with $R_0 \leq 1$. The extinction probability of a branching process epidemic with $R_0 > 1$ is given by the unique solution q on $(0,1)$ to the equation [5]

$$q = G(q). \tag{25}$$

Branching processes which go extinct are often referred to as *mortal* branching processes [6], and in epidemiological contexts the terminology *minor outbreak* is used.

To effectively control an outbreak (in the sense of guaranteeing that all outbreaks go extinct), we need to force the effective reproductive ratio R_e below one. Consider a control measure that reduces the average number of susceptible contacts made during an infectious period to $N_c < N$. This measure reduces the mean of the Poisson contact distribution while leaving the mean of the beta transmission probability intact at its value of λ/N. Under this control measure the effective reproductive ratio is

$$R_e = N_c \frac{\lambda}{N}. \tag{26}$$

Thus, R_e is below one when $N_c < N/\lambda$. Such a control measure can be achieved by instituting quarantine measures or school/work closures to reduce the number of contacts made, or through a vaccination program which reduces the number of susceptible contacts made. In the latter case, we observe that at the critical level of vaccination $N - N_c = N(1 - 1/\lambda)$, giving us the critical vaccination formula commonly found for compartmental models [7].

6 Minor outbreak size calculations

The probability that a mortal branching process with m initial particles attains a total size of Z is given by the joint probability that the Z particles generate $Z - m$ particles between them (i.e. there are as many birth events produced as there are progeny in the entire process), scaled through by a factor of m/Z. The outbreak size probability

$P(Z = z|m)$ in our branching process model is thus given by

$$P(Z = z|m) = \frac{m}{Z}\frac{G_Z^{(z-m)}(0)}{(z-m)!}$$
$$= \frac{m}{Z}\frac{1}{(z-m)!}\frac{\mathrm{d}^{z-m}}{\mathrm{d}s^{z-m}}[M(\lambda\Phi, N\Phi, N(s-1))]^z|_{s=0}. \tag{27}$$

The kth derivative of the confluent hypergeometric function $M(a, b, z)$ is given by [1]:

$$\frac{\mathrm{d}^k}{\mathrm{d}z^k}M(a, b, z) = \frac{a^{(k)}}{b^{(k)}}M(a + k, b + k, z), \tag{28}$$

and so

$$\frac{\mathrm{d}^k}{\mathrm{d}s^k}M(\lambda\Phi, N\Phi, N(s-1))|_{s=0} = \frac{(\lambda\Phi)^{(k)}}{(N\Phi)^{(k)}}M(\lambda\Phi + k, N\Phi + k, -N). \tag{29}$$

Using the Leibniz rule:

$$\frac{\mathrm{d}^n}{\mathrm{d}x^n}(f(x)g(x)) = \sum_{k=0}^{n}\binom{n}{k}f^{(k)}(x)g^{(n-k)}(x), \tag{30}$$

Equation 27 becomes

$$\begin{aligned}P(Z = z|m) &= \frac{1}{(z-m)!}\frac{\mathrm{d}^{z-m}}{\mathrm{d}s^{z-m}}M(\lambda\Phi, N\Phi, N(s-1))[M(\lambda\Phi, N\Phi, N(s-1))]^{z-1}|_{s=0} \\ &= \frac{1}{(z-m)!}\sum_{k=0}^{z-m}\binom{z-m}{k}N^k\frac{(\lambda\Phi)^{(k)}}{(N\Phi)^{(k)}}M(\lambda\Phi + k, N\Phi + k, -N)\times \\ &\quad \frac{\mathrm{d}^{z-m-k}}{\mathrm{d}s^{z-m-k}}[M(\lambda\Phi, N\Phi, N(s-1))]^{z-1}|_{s=0}.\end{aligned} \tag{31}$$

Derivatives of the form $D_{i,j} = \frac{\mathrm{d}^i}{\mathrm{d}s^i}[M(\lambda\Phi, N\Phi, N(s-1))]^j|_{s=0}$ for $i = 1, 2, 3, ...,$ $j = 1, 2, 3, ...$ can be calculated recursively using the Liebniz rule:

$$\begin{aligned}D_{ij} &= \frac{\mathrm{d}^i}{\mathrm{d}s^i}M(\lambda\Phi, N\Phi, N(s-1))[M(\lambda\Phi, N\Phi, N(s-1))]^j|_{s=0} \\ &= \sum_{k=0}^{i}\binom{i}{k}N^k\frac{(\lambda\Phi)^{(k)}}{(N\Phi)^{(k)}}M(\lambda\Phi + k, N\Phi + k, -N)\frac{\mathrm{d}^{i-k}}{\mathrm{d}s^{i-k}}[M(\lambda\Phi, N\Phi, N(s-1))]^{j-1}|_{s=0} \\ &= \sum_{k=0}^{i}\binom{i}{k}N^k\frac{(\lambda\Phi)^{(k)}}{(N\Phi)^{(k)}}M(\lambda\Phi + k, N\Phi + k, -N)D_{i-k,j-1}.\end{aligned} \tag{32}$$

The jth column of the matrix $\boldsymbol{D} = (D_{i,j})$ is expressed in terms of the $j - 1$th column. To initiate the recursive process, we note that $D_{i,1}$ is just the ith derivative of the confluent hypergeometric function $M(\lambda\Phi, N\phi, N(s-1)$ evaluated at $s = 0$,

$$D_{i,1} = N^i\frac{(\lambda\Phi)^{(i)}}{(N\Phi)^{(i)}}M(\lambda\Phi + i, N\Phi + i, -N). \tag{33}$$

To calculate the probability of obtaining an outbreak of size z from m index cases, we need to calculate the $(z - m) \times z$ submatrix $\boldsymbol{D}^{m,z}$ which restricts the matrix $\boldsymbol{D}$ to rows $i = 1, ..., z - m$ and columns $j = 1, ..., z$. This submatrix contains all the submatrices necessary for calculating all the outbreak size probabilities up to $P(Z = z|m)$, and so we effectively obtain the entire distribution up to z.

7 Major outbreak attack rate

The attack rate of a major epidemic in a large population can be closely approximated by a Gaussian distribution with mean Z_∞, which is the unique solution to the equation [5, Chapter 4]

$$\tau = 1 - \exp(-R_0\tau). \tag{34}$$

If we assume that infections are generated at the points of a Poisson process of fixed intensity $\tilde{\lambda}$ over a randomly distributed infectious period with expectation $\tilde{\lambda}$ and variance ι, and denote by Z_n the size of an outbreak in a closed population of size n, then the product $\sqrt{n}(Z_n/n - \tau)$ converges to a normal distribution with mean 0 and variance [5, Chapter 4]

$$\frac{\rho\tau + \tilde{\lambda}^2\sigma^2\tau\rho}{(1-\tilde{\lambda}\iota\rho)^2} \tag{35}$$

where $\rho = 1 - \tau$. Thus, for large population size n, the variance of the major outbreak attack rate is approximately

$$\frac{1}{n}\left(\frac{\rho\tau + \tilde{\lambda}^2\sigma^2\tau\rho}{(1-\tilde{\lambda}\iota\rho)^2}\right). \tag{36}$$

Although our model does not make any assumptions about the distribution of infectious period, it is statistically equivalent to an epidemic with intensity N over a beta-distributed infectious period with mean $\tilde{\lambda} = \lambda/N$ and variance $\iota = \frac{\lambda(N-\lambda)}{N^2(N\Phi+1)}$. When population size n is large, the major outbreak attack rate of the beta-Poisson model is thus approximately Gaussian with variance

$$\frac{1}{n}\left(\frac{\rho\tau + \lambda^2\frac{\lambda(N-\lambda)}{N^4(N\Phi+1)}\tau\rho}{(1-\lambda\rho)^2}\right). \tag{37}$$

Since the major epidemic attack rate distribution is conditional on not going extinct in the early branching stages of the epidemic, it needs to be scaled by a factor of $(1-q)$ to give the approximate unconditional probabilities of attaining these sizes.

8 Likelihood calculations using outbreak size data

By fixing an average number of contacts N, the beta-Poisson model makes explicit assumptions about underlying contact structure. This means that when fitting the model to outbreak size data we require that all of the outbreaks be from the same underlying population (or a set of populations with similar contact behaviour). For a dataset containing Z cases attributable to m importations, a maximum likelihood estimator for λ is given by [6]

$$\hat{\lambda} = 1 - \frac{m}{Z}. \tag{38}$$

This formula is accurate only when none of the importations have resulted in major outbreaks. Becker [6] outlines the calculation of an MLE when some of the outbreaks are in fact major, but this only works when the underlying offspring distribution is a power series distribution.

Using the minor outbreak size distribution to perform likelihood calculations is very inefficient, since we need to calculate all outbreak size probabilities up to the maximum in our dataset. Intuitively we would expect this distribution to be heavy-tailed, containing large outbreaks with big gaps between the outbreak sizes. This means we need to calculate lots of outbreak size probabilities which we do not directly use. One possible improvement when the underlying population is relatively small is to use the

minor outbreak size distribution for outbreak sizes below a certain threshold, and the normally distributed major attack rate distribution for outbreak sizes above that threshold. Because the branching process approximation begins to diverge from the standard epidemic model when the epidemic size reaches the square root of the population size [8], a natural threshold for a population of size M is $\sqrt{M}$. The cumulative outbreak size probability up to $\sqrt{M}$ may be substantially smaller than the extinction probability q, and so we denote this cumulative probability q_M. This is the probability of a minor outbreak in a population of size M, using the definition in terms of divergence from the standard epidemic model. The normally distributed major attack rate distribution is scaled by $(1 - q_M)$ rather than $(1 - q)$. Andersson and Britton [5] suggest that the normal approximation for major outbreaks is reasonably accurate for populations of around size 100, and so this fitting procedure is most useful for populations in the low hundreds, becoming less useful as $\sqrt{M}$ grows and we need to carry out larger and larger matrix calculations. It is important to note though that because the major outbreak attack rate always has to be between 0 and 1, this normal approximation will be less accurate when the expected attack rate is close to either of these extremes since the true distribution can not have symmetric tails in this case.

9 Fitted parameter values

Parameter fits for the negative binomial and ZIP models are provided in Tables A and B. Fits and confidence intervals for the parameter λ in the Poisson and geometric models are identical to those for λ in the negative binomial and beta-Poisson models and so are not listed here.

**Dataset**	λ	θ
Plague	1.32 (0.88, 1.82)	0.92 (0.12, 1.98)
Mpox	0.3 (0.22, 0.4)	0.52 (0.1, 1.05)
Ebola, Nigeria 2014	0.95 (0.15, 2.3)	6.89 (0.0, 24.55)
Ebola, Guinea 2014	0.95 (0.61, 1.36)	5.26 (2.66, 8.77)
SARS, Singapore 2003	0.79 (0.36, 1.38)	8.5 (2.07, 18.87)
MERS, South Korea 2015	1.05 (0.2, 2.31)	28.18 (1.91, 77.98)
MERS, Saudi Arabia 2015	0.96 (0.42, 1.67)	1.33 (0.0, 4.0)
Norovirus, Netherlands 2012	0.93 (0.61, 1.3)	0.56 (0.0, 1.39)

Table A. Maximum likelihood estimates of negative binomial model parameters by dataset, 95% confidence intervals in parentheses.

**Dataset**	$\tilde{\lambda}$	σ
Plague	1.87 (1.15, 2.6)	0.29 (0.05, 0.48)
Mpox	0.72 (0.37, 1.09)	0.58 (0.28, 0.72)
Ebola, Nigeria 2014	3.71 (0.15, 8.33)	0.74 (0.0, 0.9)
Ebola, Guinea 2014	3.24 (2.12, 4.46)	0.71 (0.62, 0.78)
SARS, Singapore 2003	4.0 (1.63, 6.8)	0.8 (0.73, 0.86)
MERS, South Korea 2015	8.7 (1.47, 18.67)	0.88 (0.82, 0.92)
MERS, Saudi Arabia 2015	1.71 (0.61, 3.13)	0.44 (0.0, 0.68)
Norovirus, Netherlands 2012	1.31 (0.73, 1.93)	0.28 (0.0, 0.5)

Table B. Maximum likelihood estimates of zero-inflated Poisson model parameters by dataset, 95% confidence intervals in parentheses.

10 Sensitivity analysis

To better understand the fitting behaviour of the beta-Poisson model we calculate one-dimensional cross-sections of the likelihood surface associated with each dataset. In each case we calculate each of the beta-Poisson parameters λ, Φ, and ν over a range of values with the other two parameters fixed at their MLEs. These cross-sections are plotted in Fig A.

The plots in the middle column of Fig A reveal substantially different fitting behaviour for the parameter Φ depending on the level of overdispersion seen in the dataset in question. Fig A a)(ii), b)(ii), g)(ii), and h)(ii), corresponding respectively to the plague, Mpox, Saudi Arabian MERS and norovirus datasets (all with low levels of overdispersion) all show a comparatively gentle decrease in likelihood as Φ increases away from its MLE, whereas Fig A c)(ii), d)(ii), e)(ii), and f)(ii), corresponding to the more overdispersed Nigerian Ebola, Guinean Ebola, SARS, and South Korean MERS datasets, all display a much more defined peak around the MLE. The third column shows a high likelihood assigned to $\nu = 0$ for all the datasets, supporting our finding that the beta-Poisson is unable to offer a substantial improvement in likelihood over the negative binomial model.

References

1. Abramowitz M, Stegun IA. Handbook of mathematical functions: with formulas, graphs, and mathematical tables. vol. 55. Courier Corporation; 1965.

2. Karlis D, Xekalaki E. Mixed poisson distributions. International Statistical Review. 2005;73(1):35–58.

3. European Mathematical Society. CConfluent hypergeometric function. In: Encyclopedia of Mathematics. EMS Press; 2020.

4. Dion JP. Estimation of the mean and the initial probabilities of a branching process. Journal of Applied Probability. 1974;11(4):687–694.

5. Andersson H, Britton T. Stochastic epidemic models and their statistical analysis. vol. 151. Springer Science & Business Media; 2012.

6. Becker N. On parametric estimation for mortal branching processes. Biometrika. 1974;61(2):393–399.

7. Keeling MJ, Rohani P. Modeling Infectious Diseases in Humans and Animals. Princeton University Press; 2007.

8. Ball F, Mollison D, Scalia-Tomba G. Epidemics with two levels of mixing. Ann Appl Probab. 1997;7(1):46–89. doi:10.1214/aoap/1034625252.

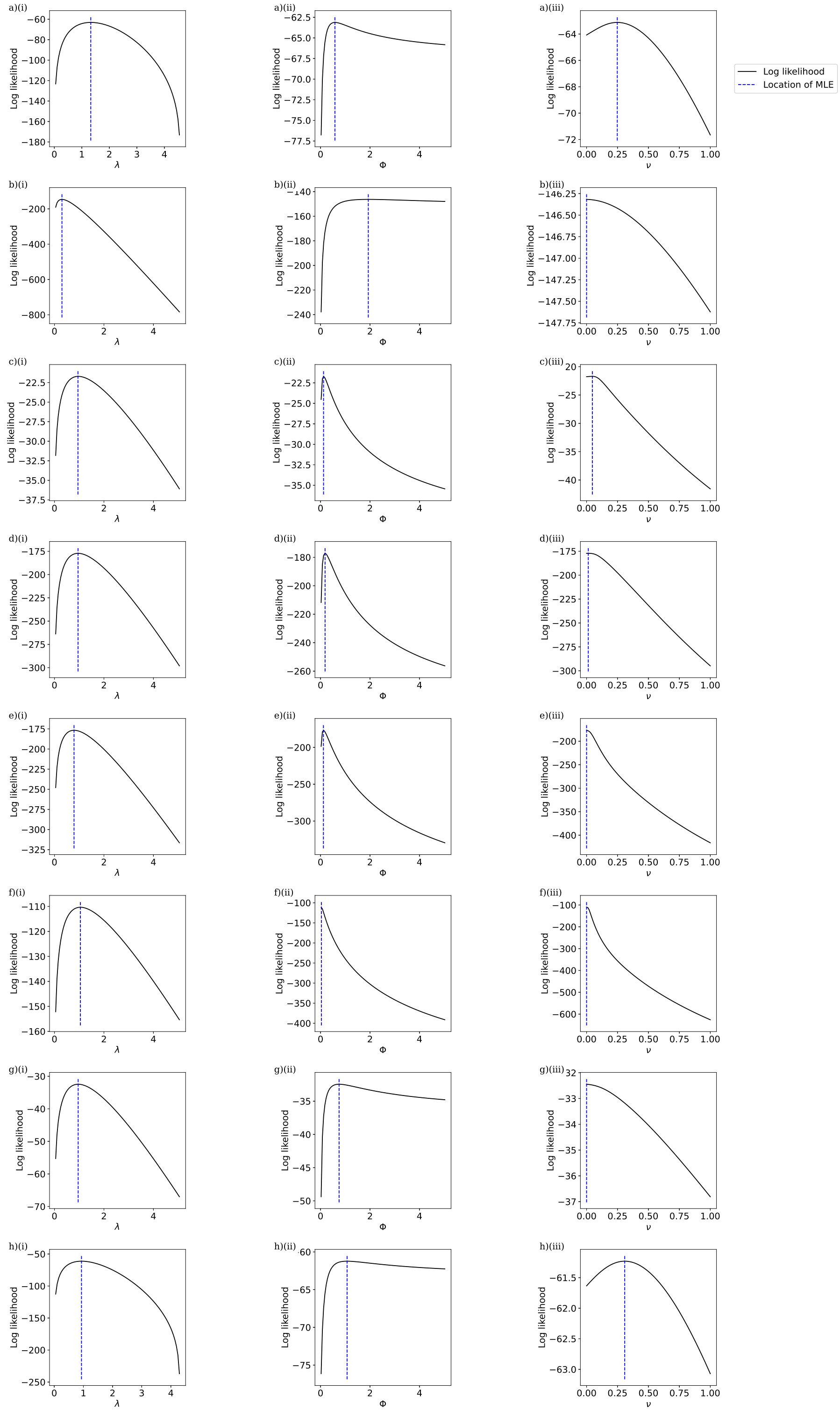

Fig A. Log-likelihood curves of the beta-Poisson model parameters by dataset. Row a) plague; row b) mpox; row c) Ebola, Nigeria 2014; row d) Ebola, Guinea 2014; row e) SARS, Singapore 2003; row f) MERS, South Korea 2015; row g) MERS, Saudi Arabia 2015; row h) norovirus, the Netherlands 2012; column (i) log-likelihood of λ values with Φ and ν fixed at MLEs; column (ii) log-likelihood of Φ values with λ and ν fixed at MLEs; column (iii) log-likelihood of ν values with λ and Φ fixed at MLEs. MLEs marked at zero in column (iii) correspond to cases where the beta-Poisson achieves maximum likelihood at its negative binomial limit.
