# Supplementary material for: A beta-Poisson model for infectious disease transmission
Source: PLoS Comput Biol. 2024 Feb 8;20(2):e1011856. doi: 10.1371/journal.pcbi.1011856 (PMC10903957; doi:10.1371/journal.pcbi.1011856)
Supplement: Attachment — Submitted filename: response-to-reviewers.docx [file pcbi.1011856.s002.docx]

Dear editors

We would like to thank yourselves and the reviewers for their constructive feedback, which we have taken into careful consideration when creating our revised manuscript.

In recognition of the comments from all three of the reviewers on the manuscript’s structure, we have substantially restructured the manuscript to improve clarity and narrative flow. In line with the paper’s focus on methodology, we have rearranged the sections of the manuscript so that the Methods now comes between the Introduction and Results sections. We have moved most of the mathematical details from the Methods section of the main paper to the Appendix, where this content forms Sections 1 and 2 (“Model description” and “Likelihood calculations using secondary case data”). The Methods section of the main paper now covers the basic conceptual structure of the beta-Poisson model, explains the methods used to carry out our likelihood-based analysis, and gives a description of the datasets used in our analysis. Much of this content was covered in the Results section of our original submission. The Results section of our revised manuscript focusses solely on the results of our likelihood-based analysis, with all the methodological details covered in the Methods section.

The other consistent theme in the feedback we received was the lack of a clear motivation for the study. Our interest in the proposed beta-Poisson model stems from its potential application in modelling non-pharmaceutical interventions; by modifying the model’s social contact parameter and keeping the parameters of the underlying beta distribution fixed we can project the impact of interventions which change contact rates while preserving individual-level variation in infectiousness. With this in mind we have heavily revised the Introduction and Discussion to emphasise this motivation.

Our response to the specific comments left by the reviewers are below.

Best wishes

Ian Hall and Joe Hilton

**Reviewer 1**

*“While the authors nicely summarize the current practices and backgrounds regarding modelling of overdispersion until L67, the following transition to the beta-Poisson mixture, the topic of this paper, reads rather abrupt and awkward. In particular, the motivation for this study (i.e., why the authors thought the current approaches may be insufficient and were interested in beta-Poisson) should be provided.”*

We have revised this transition (L80 onwards) to emphasise the motivation for the beta-Poisson model as a more mechanistically interpretable alternative to other Poisson mixture models, and its potential applicability in modelling control measures which reduce social contact rates.

“*I also did not fully get the objectives of the paper from reading the introduction section. Either the “effectiveness” inL83 or “performance” in L85 was not defined and I do not know what the authors mean here.*”

We have revised the last paragraph of the introduction (L102-113) to specify that we are comparing the beta-Poisson model to the Poisson, geometric, negative binomial, and ZIP models in terms of goodness-of-fit and ability to capture specific metrics of the observed offspring distribution.

“*Results: I see this section is not really pure results and the authors explain a lot of background and methods along with the results, which I feel is making it challenging for the readers to take in. Meanwhile, the current method section contains obvious demonstrations that aren’t necessarily crucial in the main text. For example, it’s nice that the authors provide step-by-step explanation of how the beta-Poisson model can be constructed, but a typical reader of PLoS Comp Bio would know what it is just from reading L75–77 or L92–95. I would suggest the current method section is moved to the supplementary materials entirely and the methodological explanations in the current results section are instead moved to the method section. That way, the readers would better understand what the authors did in this paper, rather than each mathematical concept that appears in the methodology. On that note, it would also be helpful to split the results section into multiple subsections*.”

As we have detailed above, we have revised the structure of our manuscript in line with these suggestions.

“*As noted above, Kremer et al. has studied a similar (though with a different interest and direction) topic. The authors may not have been aware of this paper as it is not included in the reference list, but I think it would potentially inform how the authors should frame this paper*.”

We had not seen this paper and thank the reviewer for pointing us towards it. While the Kremer et al. study concerns a similar modelling approach and type of data, their proposed Poisson-generalised gamma mixture model does not allow for an interpretation in terms of a two-step contact-and-infection process, which is the main conceptual advantage of our beta-Poisson mixture approach. We have added a brief reference to the study as an exploration of alternatives to the negative binomial in our Introduction (L95-101). The Kremer et al. study finds that two-parameter subcases are consistently preferable in terms of goodness-of-fit to their more general three-parameter model, which is analogous to the result which we obtain, and we have mentioned this similarity in outcome in our Discussion (L329-333).

“*L9–13: I am not sure if this is really the case in real-world outbreaks. There may well be overlooked cases in the earliest phase of a spillover event; contact tracing could also be challenging. Do the authors have any evidence supporting their statement?”*

In our original manuscript we claimed that outbreaks of novel pathogens typically allowed for more complete observation of transmission events than larger outbreaks, which we agree may be an overly optimistic claim. We have removed this claim in favour of a brief explanation of the usefulness of contact tracing and its role in generating the type of data we use in this study(L15-23).

*“L14: “its mean”: I don’t get what mean is being discussed here”*

Here we meant the mean of the network obtained by drawing edges from each case to its secondary cases; we have revised this sentence to make this more clear (L23-26).

*“L88–89: this would be better place at the end of the methods section.”*

As suggested the link to our Github repo has been moved to the end of the Methods section.

**Reviewer 2**

“*One of the overall findings of the paper is that even though the beta-Poisson achieves a closer fit to the data, in most cases the negative binomial outperforms the beta-Poisson in terms of the AIC. This is stated in the abstract and in lines 203-205 in the Results when referring to Table 2. I think in an attempt not to oversell their results, the authors conclude that the beta-Poisson is never the optimal choice. This is true, but the difference in AIC values in Table 2between the negative binomial and the beta-Poisson never exceeds 2, and Table 4 shows that the likelihood ratios are not significant. It really comes down to the extra parameter in the beta-Poisson. The beta-Poisson also consistently outperforms the Poisson, geometric, and ZIP distributions, and these distributions are commonly used. It might be appropriate to reserve the beta-Poisson for special cases, but I would like to see the authors take a stronger position in favor of the beta-Poisson and give more reasoning and concrete examples of why the beta-Poisson is interesting and useful in the context of intervention modeling. I think the beta-Poisson’s inclusion of a social contact parameter is interesting and important, and the way in which this arises in the calculation of the extinction probability in section 3 of the supplement seems very useful.*”

As mentioned above, the paragraph starting around L80 in the Introduction emphasies the beta-Poisson model’s motivation as a potential model for interventions. We have added a mention of the extinction probability result to the Methods section of the main paper (L160-166) which we hope emphasises the potential applicability of our approach. We restate this potential at the beginning of our Discussion section (L306-312).

*“The fact that the beta-Poisson distribution has three parameters, makes this distribution more cumbersome than the negative binomial, and in some cases the overdispersion parameter, phi, is unidentifiable. This is evident in the fitting behavior presented in the supplement. The fact that phi is hard to identify does not seem surprising since this parameter always appears as a product of either lambda or N. This could be why your 95% CIs in Table 3 are wide for phi. This phenomenon should be addressed/discussed in the manuscript. In addition, if you use the formulation of the beta-Poisson as it is written in Equation 1 in the main text and find the MLEs alpha_1, alpha_2, and N for the data, does this provide a better fit than making the substitution for phi? Would it be possible to fit the data and make the substitution for phi after obtaining the MLEs?”*

Based on this suggestion we have tried repeating our analysis with a few different parameterisations of the beta-Poisson model. Using an (N, alpha_1, alpha_2) formulation resulted in very wide confidence intervals for alpha_2. As the reviewer has pointed out for the parameter Phi, this makes sense since alpha_2 only ever appears in a sum with alpha_1, with that sum itself only appearing as an argument to the confluent hypergeometric function. As an alternative we also considered defining Phi=(alpha_1+alpha_2) rather than Phi=(alpha_1+alpha_2)/N, but with this formulation we lose the natural interpretation of Phi as the inverse of the overdispersion of the limiting negative binomial distribution. Based on these results we have opted to retain the (lambda, Phi, N) formulation used in our original submission. The study by Kremer et al. mentioned by Reviewer 1 is possibly relevant here – they find that two-parameter subcases consistently outperform a three-parameter general case in terms of goodness-of-fit to secondary case data, possibly suggesting that secondary case numbers simply aren’t informative enough to confidently infer three model parameters.

*“There needs to be a stronger concluding paragraph or section that sums up the take-home message and keyfindings. The reader is left wanting more at the end of the discussion”*

We have revised our Discussion section and have removed much of the extraneous discussion covering general aspects of parameter fitting in infectious disease dynamics. The revised Discussion section is much more focussed on the beta-Poisson model: in the first paragraph (L298-329) we emphasise the beta-Poisson’s motivation in terms of intervention modelling while recapitulating the findings from the Results section which suggest that it is not a substantial improvement over the negative binomial in terms of goodness of fit. This is followed by a second paragraph (L334-364) where we discuss the implications of including an explicit social contact parameter and emphasise that care needs to be taken in extrapolating a model fitted to outbreak data from one population to other populations.

“*In the third paragraph of the introduction around line 47, it would be very helpful to have specific examples with citations of how these distributions have been incorporated in SIR models*.”

We have added a reference here to Llloyd 2001 on on infectious period distributions and epidemic persistence (<https://doi.org/10.1006/tpbi.2001.1525>) which specifically discusses the link between gamma-distributed infectious periods and multi-phase SIR models (L57-62).

*“Understanding superspreading events is one of the goals of fitting these models to data. It think it would be worth mentioning superspreading events in the first paragraph of the introduction when you are explaining the processes that are important to understanding transmission events.”*

Superspreading is now discussed in the first paragraph on L4-15.

*“Line 97 and Line 199: generalisation should be generalization.”*

This has been corrected.

*“Line 121-124: Maybe I am wrong, but I think that the beta-Poisson distribution can be valuable in more contexts than just being a model that “fits the data better.” For example, the beta-Poisson might be valuable in simulation-based modeling to generate this type of transmission chain.”*

In the Methods section around L160-176 we now emphasise that the potential value of the beta-Poisson model is indeed in intervention modelling, and that we are performing model fits to assess whether it is *also* an improvement over its subcases in terms of goodness-of-fit.

*“Line 129: Which algorithm did you use in this package to find MLEs? This should be stated in this sentence.”*

In the paragraph beginning L177 in the Methods section we now specify that we are using the implementation of the BFGS algorithm for finding extrema in the minimize function from the scipy.optimize package in Python.

*“Figure 1: I really like this figure. It is an excellent depiction of how each of the distributions model transmission. The graphs in each panel need to be defined. In the negative binomial panel, for example, the function with the solid line represents the gamma distribution and the dotted function represents the secondary cases. It would be very helpful if these were labeled (same with the beta-Poisson panel). In the last panel on the beta-Poisson, the individuals without the disease are confusing to the reader because it looks like the disease chain stops. I think it would be clearer if the authors kept only the two individuals where one arrow went to the secondary infected individual with probability p and the other arrow when to the uninfected contact.”*

We have added labels to the distribution plots and have simplified the beta-Poisson schematic as suggested.

*“Line 187-188: It would be helpful if you use the term “superspreading boundary” in this sentence so we have a definition for this term when it appears in the caption for Table 1.”*

This definition now appears on L244-248 when this table is referenced (note that this is now Table 2 rather than Table 1).

*“Table 4: It would be helpful to add degrees of freedom for the likelihood ratios in this table so they can be interpreted using Wilk’s theorem.”*

The difference in number of parameters between each model and the beta-Poisson is now listed in brackets after the name of each distribution.

*“I am not sure about the formatting requirements of the journal, but I do think the paper might be clearer if the methods section with a description of the model and the data sets came before the results and the discussion.”*

This is covered by our revisions to the manuscript structure.

*“The parameter symbols and their definitions are hard to keep track of. For example, the authors use several terms that describe a measure of overdispersion (phi in line 91 and theta=1/phi in line104), and N and nu=1/N in line 137. It would be helpful to have a table containing all the parameters, their symbols and their definitions to accompany the model description.”*

*“It would also be helpful to have a table of the five distributions that you are comparing that contains the parameters that are present in each model and states how many parameters in each model are being estimated. This would greatly help with the AIC interpretation.”*

To minimise the proliferation of tables, our revised manuscript includes a single table (Table 1) listing the paramaters of each model, including notation, interpretation, and possible values (which should help in interpreting fitting results). In Table 3, where we list the AIC obtained in each model-dataset combination, we have added the number of parameters to the column headers in brackets after the name of each model.

*“Line 209: I am confused here about why you would reject the geometric distribution. I would interpret the results in Table 4 to mean that the goodness of fit is indistinguishable between the different models. This should be clarified.”*

Thank you for catching this – this was a mistake on our part. We meant to say that the geometric distribution is optimal in terms of AIC and also can not be rejected based on the likelihood ratio. The text has been updated accordingly (L269-273).

*“Line 238: “The beta-Poisson distribution will be definition achieve a closer fit to transmission chain data…..” why is this true?”*

We now emphasise here that this is because the beta-Poisson is a generalization of the negative binomial (L305-308)

*“Line 240: “but our results demonstrate that these models outperform it equally consistently on grounds of parsimony. This sentence is very confusion and needs to be revised. What are “these models” and what is “it”?”*

This sentence in hindsight was very unclear. What we meant specifically was that certain subcases of the beta-Poisson are likely to be chosen over it on the basis of AIC given a set of secondary case data. The first paragraph of our revised Discussion section (L332-334) hopefully expresses this general point more clearly.

*“Line 241: Would there ever be a case when the topology of transmission chains is NOT simple enough to be summarized by two parameters?”*

Given the similar findings in the Kremer et al. study on Poisson-generalised gamma mixtures, it seems likely that secondary case data of the type we have considered (i.e. without detailed data on patient characteristics which could inform a multitype branching process model) will generally not be rich enough to confidently inform a three-parameter model. We have added a reference to this study to this effect on L329-333 of the Discussion.

*“Line 275: The parameters lambda and theta have taken on different definitions throughout the paper. Define them again here in this sentence.”*

In revising the Discussion section of the manuscript we have removed the reference to these parameters entirely.

*“In Figure A in Section 8 of the supplement, I do not understand why nu = 0 in the third column. In the figure caption it says that nu is fit to the MLE. This should be clarified.”*

The case nu=0 corresponds to cases where the MLE is at the beta-Poisson’s negative binomial limit. We have added an explanation to this effect to the caption of this Figure.

**Reviewer 3**

We hope that the structural revisions mentioned at the start of this response will be sufficient to address the comments on manuscript organisation at the start of the reviewer’s comments.

“l.14 "its mean": re-word around this phrase. This seems to refer to the mean of the network, which is not a well-defined concept. I think you're trying to refer to the mean of the offspring distribution of the Branching Process implied by the network structure, but that's probably quite hard to explain this early in the manuscript?”

We have revised this phrasing to make it clear that we are talking about the mean number of onward connections from each case in the transmission network, and have placed this in the context of a discussion of contact tracing (L19-26).

“l.25: The word "assigned" here probably needs some explanation.”

We have replaced “assigned” with the less ambiguous “traced back” (L36).

“l27-28: The phrase "outbreak size distribution" might need explanation too. The phrasing perhaps implies final outcome, but looking at "before depleted" on l.19 it might also be restricting attention to outbreaks which are in some sense small compared to the population size?”

We have rephrased this (L37-39) more precisely: “Results from the theory of branching processes can be used to calculate useful quantities like the probability that an outbreak goes extinct and the probability that it reaches a given size before extinction”.

“l.44 "continuous". Usually the mixing distribution is continuous, but not necessarily.”

This is a very good point. We have removed the word “continuous”.

“l.95: I assume that each of these contacts which are infected with probability p are infected independently? The independence (given p) is crucial to spell out so the model is clear to the reader. I suggest making this clear by including (preferably here, but I'm a mathematician so elsewhere might actually be more appropriate) the accompanying definition of a beta-Pois variable Y as (Y|X=x,P=p) \sim Bin(X,P), where X \sim Poi(N) and P \simBeta(a1,a2); with the note that this is equivalent to Y|P=p \sim Poi(N \cdot p), where P \sim Beta(a1,a2). This (i)explicitly connects two versions of the distribution (which I don't think is done at present), the former being nicer for interpretation as done around l.95 and the latter being a little mathamatically simpler and thus nicer for calculations and (ii) makes clear from the latter version that it is a mixed-Poisson distribution. Because of the representation Y|P=p\sim Poi(N \cdot p), I wonder if it might be more informative to call this a scaled-Beta-Poisson distribution; since your version uses a scaled Beta random variable (N \dot P) instead of a Beta random variable (just P) as the mixing distribution for the Poisson variable, i.e. as the distribution for the mean of the conditional Poisson variable.”

This was a really useful suggestion; our original manuscript lacked an explicit explanation of the beta-Poisson-binomial to beta-Poisson reasoning. This explanation now forms the first paragraph of our revised Methods section (L116-136).

“l.124: What is meant by "their fitting behaviour"?”

We now make it clear that we are assessing the goodness of fit of each model, and the ability to capture specific epidemiological characteristics (proportion of superspreaders, proportion of cases which generate no onward infection, overdispersion of secondary case distribution) (L169-177).

“l.126: What is a confidence interval for a model fit? Do you mean "CIs for these parameters"? Also I do not understand what "a grid calculation" means; please either explain or give a reference.”

We have clarified that we are referring to the confidence intervals of the parameters (L190-193). The mention of a grid calculation was a mistake – in an earlier draft we discussed the calculation of the full likelihood surface here as well as the confidence intervals, and the grid calculation was used to calculate this surface. We have corrected this to specify that the confidence intervals are calculated by bootstrapping (L192-193).

“l.129: Presumably the CIs are symmetric in the sense of having equal probability in each tail?”

This is correct, we now specify that they are symmetric (L190).

“l.137: Is it possible to interpret the parameter nu? That would be nice for the reader, even if it's only a bit vague /hand-wavy.”

Here (L187-190) and elsewhere (in particular Table 1) we now refer to it as an “inverse contact parameter”.

“l.190: I think this sentence would be more precise/accurate if "record the number of" is changed to "make up the". It also hinders understanding that the completely different phrasing "superspreading boundary" is used for what seems to be the same thing in the caption for Table 1.”

We have adopted the suggested phrasing (L248) and have also introduced a definition for the terminology “superspreading boundary” (L245-249).

“Table 1 & Table 2 highlight particular entries with different methods (bold v underlining). Suggest consistency. It might also be worth mentioning in the discussion that actually several rows of Table 2 have multiple entries quite close to the minimum entry, so which is the "best" model is not clear-cut for some datasets.”

We have changed the bolding in Table 1 to underlining for consistency.

“l.201-202: I don't know what to understand from the phrasing "if another distribution appeared to offer a higher maximum likelihood". Surely if the beta-Poiss is more general then this is impossible?”

The point we were trying to make here was precisely that this is impossible, but we recognise that our phrasing was not very clear. We have rephrased to instead emphasise that any likelihood available to one of the subcase models is also available to the beta-Poisson through an appropriate parameter choice (L264-268).

“l.213: I don't know what it means for a model to fit "decisively".”

Here we meant that the confidence intervals around the inverse contact parameter nu were very narrow, and we have rephrased to say exactly this (L279-280).

“l.126 "and in these cases": suggest a new sentence here, the sentence is quite long.”

We have revised this sentence into a single more concise sentence (L277-280).

“Table 3: What do the entries 0.0 mean? Is this just rounding for display? Need to make this clear (or explain/remindwhy/how the MLE / CI endpoint can be exactly zero).”

For the parameter Phi these are indeed rounded for display purposes, whereas for the parameter nu these represent “true” zeros where the maximum likelihood is attained at the negative binomial limit nu=0. We have added a clarification on this to the caption of Table 4 (Table 3 in the original submission).

“l.220: This type of explanatory sentence is missing for many other Figures/Tables.”

We have added a sentence specifying that the same outputs are plotted for the beta-Poisson and negative binomial models in cases where the beta-Poisson fits to its negative binomial limit (L297-300)..

“l.227/232/235: Why present a figure and a table with the same information in? Should choose one. I slightly prefer thefigures, subject to the comment below about Figure 2.”

We have removed the Tables of overdispersion, proportion of superspreaders, and proportion of zeros in favour of figures.

“l.227-228: Not clear to me what "a distinct distribution" means here. Maybe one of the special cases mentioned before? If so, this is not really distinct as the beta-Pois family includes these special cases.”

We here meant “distinct from the negative binomial limit”, i.e. a parameterisation of the beta-Poisson with nu>0. We have rephrased to make this more clear (L285-286).

**The following comments all refer to the Discussion section of the paper, which has been substantially rewritten based on feedback from all three reviewers. Given the scale of these revisions we can not point to an exact line-for-line replacement to the words and phrasings mentioned here in the revised manuscript:**

- “l.238: I don't understand the use of "consistently" here. Surely "always" is correct since beta-Poi is more general thant hose more common distributions?”

We agree that using “always” in favour of “consistently” here would be correct.

- “l.243: unidentifiability seems to come out of nowhere for me; not mentioned in the results section I don't think.”

Rather than talk about unidentifiability, we now talk specifically about wide confidence intervals on the parameter Phi (L314-316), which avoids bringing in new concepts so late in the manuscript.

- “l.246: "but clearly not infinite" is a phrasing I don't understand: a real-valued parameter cannot be infinite.”

Here we meant that the likelihood of the beta-Poisson parameters was maximised with a large contact rate but not at the negative binomial limit.

- “l.251: Not clear to me what "Poisson behaviour" means here.”

We here meant that social contacts are assumed do be drawn from a Poisson distribution. We have rephrased this point more precisely on L321-324.

- “l.261: Does "outbreak size" here refer just to the size of small outbreaks?”

This is correct. We have moved this mention of outbreak size calculations to the Methods section (L194-201) and specified there that we are talking about epidemics which die out in their early branching process phase.

- “l.283: "is" and "implications" plural.singular mismatch.””; “l.305: "is is" should I think be "it is".”

The sentences containing these mistakes no longer appear in the revised Discussion section.

- “l.308: "contact survey also suggests" needs rewording, perhaps "contact surveys also suggest" is meant?”

This has been corrected (L347)

“l.339: To be precise I think this should be "their total number of contacts during this time follows a Poission distribution with mean N".”

This has been rephrased accordingly (Appendix L12-13)

“l.341-342: Independence for different contacts key to mention here.”

We now specify this (L15).

“Display (1): Suggest in the first line leaving e^{-pN} next to (pN)^x / Gamma(x+1) so that the structure P(x|p) P(p) is more obvious. Also this equation needs to have possible values of x indicated.”

We have reformatted the first line accordingly (Appendix Display (1)) and specified that x is a non-negative integer (Appendix L19).

“Display (3): This follows with much easier calculations using the tower property. Depending on formulation used,either of these does that:

P \sim Beta(a1,a2), Y \sim Poi(N), P & Y independent; X|Y,P \sim Bin(Y,P). So E[X] = E[ E[X|Y,P] ] = E[ YP ] = E[Y]E[P] = N a1/(a1+a2). (the second-last inequality being true because P & Y are independent).

P \sim Beta(a1,a2), Y|P \sim Poi(N \dot P). So E[Y] = E[ E[Y|P] ] = E[ N \dot P ] = N E[P] = N a1/(a1+a2).

(If the equivalence of these formulations has been made explicit then the latter one is probably preferable as it is simpler.)”

This was a great suggestion and we have revised the derivation accordingly (Appendix Display (2)).

“Display (6): This will follow much more simply using the Law of Total Variance.”

Again, we have revised accordingly (Appendix Display (5)).

“l.390: "transmission distribution" phrase not used before I don't think; why introduce it here?”

Rephrased to “offspring distribution”, which is used throughout the manuscript (Appendix L66).

“l.393: Is it made explicit what (x_1, \dots, x_K) means here?”

We have specified that this is number of secondary cases per case (Appendix L69-71).

“Display (5): Please offer some explanation re where this comes from.”

We have expanding the working here to derive this explicitly from the beta-Poisson pdf (Appendix Display(8)).

“l.395: If the likelihood is not differentiable w.r.t. some of its parameters (it would be good to give a reference for this), then does this not raise lots of questions about the existence and/or uniqueness of MLEs?”

This was a mistake on our part – the confluent hypergeometric function is differentiable in each of its arguments so long as they are all positive (https://encyclopediaofmath.org/index.php?title=Confluent_hypergeometric_function), but in practice it is still easier to find the MLEs analytically. We have corrected this in Appendix L73-77.

“l.397: The comment "the branching process structure of our model means .." needs more explanation (or maybe a reference to something similar) to make it clear to the reader what the logic is here.”

We were referring here to a result on the MLE of the mean of a Galton Watson process, and have provided a brief explanation of how we apply this result (Appendix L77-87).

“l.399: What sort of numerical methods can be employed - the non-differentiability mentioned above will presumably impact this?”

In the main paper L178-190 we specify the Python function and algorithm we are using. As mentioned above, we misphrased the statement about non-differentiability – the issue is about the difficulty of easily obtaining closed form derivatives rather than differentiability as such.

“Figure 2: Why is a bar chart used here? You are not plotting a quantity of something which a bar chart naturally lends itself to. To me a point/symbol for the MLEs, with error bars as they are, would be a much more natural way to visualise this information. Also I don't think the phrase "inverse contact parameter" is used elsewhere in the manuscript.

Figs 4-6: As above, though a horizontal line could be used for the data where appropriate, so it is represented in a different way to the MLEs/CIs.”

This is an excellent suggestion. We have replotted as points for the MLEs and lines for the confidence intervals. As mentioned above we now define nu to be the inverse contact parameter when it is introduced in the main manuscript(L187-189).

“Fig 3: Suggest using bigger symbols and thicker lines so the plots are easier to read.”

We have replotted as suggested, although given some of the lines are very close together there was a limit to how thick we could make them while retaining legibility.

In Supp Mat:

“Displays (19) and (20) have variables i on the left hand side and k on the right; these should be the same presumably?”

This has been corrected (Appendix Displays (28) and (29)).

“p.5 l.1: The matrix \bold{D} = (D_{i,j}) should have a range of values specified for i & j.”

These have been specified (Appendix L155-156).

“p.5 l.6: It is not specified which elements of \bold{D} that \bold{D}^{m,z} contains.”
This has now been specified (Appendix L160-162).

“p.5 just after display (26): "This formula describes a Poisson process" is not correct, and I don't see what it might be meant to mean. A Poisson process is a random process, not a formula which can be evaluated to give a real number.”

Our phrasing was very unclear, and the notation used at the end of this sentence was also incorrect. What we meant is that the formula describes the distribution in attack rates for a model of infection where infections are generated at the points of a Poisson process over a randomly distributed infectious period. We have revised the phrasing accordingly (Appendix L171-173).

“p.6 last sentence of Sec 6: Re normal approximation for major outbreaks for populations of size 100+, this depends a lot of what tau = Z_\infty is. Broadly speaking, if tau is close to zero or one then the population size will have to be larger for the normal approximation to be reasonable; but even then how reasonable it is will depend on the particular parameters of the model.”

Thanks for pointing this out – it’s an important point and we have added a clarification to this effect on L212-216 of the Appendix.
