# Supplementary material for: A beta-Poisson model for infectious disease transmission
Source: PLoS Comput Biol. 2024 Feb 8;20(2):e1011856. doi: 10.1371/journal.pcbi.1011856 (PMC10903957; doi:10.1371/journal.pcbi.1011856)
Supplement: Attachment — Submitted filename: round2-response.docx [file pcbi.1011856.s003.docx]

Dear editors

We thank the reviewers for this secondary round of feedback, which has helped our manuscript to more fully reach its potential in terms of both legibility and overall scientific quality. Our responses to the specific comments from Reviewers 2 and 3 are listed below.

Best wishes

Joe Hilton and Ian Hall

**Reviewer #2**

*L 125 Typo here: “Each case draws an individual-level transmission probability p is drawn from from a”. “draws” and “drawn” appear in the same sentence and double “from”.*

This sentence has been revised as follows: “Each case draws an individual-level transmission probability p from a…”

*L319 Comma after interventions*

We have added the suggested comma.

**Reviewer #3**

*l125. Check wording that uses both "draws" and "drawn" in the one sentence to refer to one "drawing"; also repeated word "from". Possibly just delete "is drawn from".*

This sentence has been revised as follows: “Each case draws an individual-level transmission probability p from a…”

*l132. Check wording "rather without".*

The superfluous “without” has been removed here.

*l185. "is given by the sample mean" here seems to be a repeat of the information on l178. Given the detail presented of all the different cases I suggest deleting that first sentence of the paragraph and just presenting the discussion one model at a time.*

In line with the reviewer’s suggestion we have lightly revised this paragraph to discuss each model in sequence.

*l190. "report" might be a better word here than "generate"?*

We agree, and have reworded in line with the reviewer’s suggestion.

*l194. Suggest adding comma after "transmission chain data".*

The suggested comma has been added.

*ll205--206. Suggest rewording "x secondary cases, for x running ... to any one individual" to avoid the complicated wording involved in the precision here; perhaps to "various numbers of secondary cases"*

We have revised this sentence to read “Each of the datasets lists the number of cases in an outbreak which are inferred to have generated a given number of secondary cases”.

In revising this, we noticed incidentally that at the start of the model description we used $x$ for the Poisson-distributed number of contacts in the beta-Poisson model but have elsewhere used $x$ for the number of secondary cases generated by a single case. For consistency we have changed the $x$ at the start of the model description to a $y$ so that $x$ is reserved for secondary case numbers.

*ll208--210. First mention is of "three from ... coronaviruses", then "the two coronaviruses". If my understanding is correct it might be a bit clearer to remove the word "two" to reduce some possible confusion?*

We agree that this has the potential for confusion. We have replaced the word “two” with “novel” so that the relevant section of text reads “pneumonic plague and the novel coronaviruses are spread through airborne transmission”; this is intended to emphasise that we are referring only to the viruses covered by our datasets and not coronaviruses in general (which include some faecal-oral route infections).

*ll247--248. Suggest rewording to "... the 99th percentile of the Poisson distribution with mean matched to the dataset ,so that ..." for improved readability.*

We agree that this more concise wording improves readability, and have revised accordingly.

*l248. "correspond to" might be slightly clearer wording than "make up the" superspreading events?*

We have adopted this wording.

*Table 2 caption: "in bold" needs correcting.*

Corrected to “Underlined entries…”.

*l255. Suggest using "necessitates" rather than the past tense "necessitated".*

We agree and have changed to “necessitates”.

*l256. Missing LaTeX backslash for the Greek character nu.*

We have added the missing backslash.

*Table 3. (i) suggest aligning column entries using the decimal point for better readability. (ii) Re the comments about zeros in the caption, might it be possible/simpler to use 0.00 for rounded and 0 for true throughout the table?*

While we agree with point (i), this proved surprisingly difficult to implement in LaTeX and we have left the alignment unchanged.

Point (ii): this is a great idea, and we have accordingly changed the 0.00’s to integer 0’s in the \nu column.

*l268. Suggest "... reveals that the beta-Poisson is never ..." instead of "reveals that is never" for clarity.*

This was a typo and has been reworded as suggested.

*l269. Suggest rewording "substantial" to "substantial enough", "large enough", "enough" or similar.*

This is also a typo, and has been reworded to “substantial enough”.

*Table 5 seems very hard to interpret to me. Maybe include corresponding p-values too?*

In the first two drafts of this manuscript, Table 5 listed likelihood ratios. We have replaced this by a table of likelihood ratio test statistics which can then be directly compared with chosen significance levels for hypothesis testing.

*l300. Check repetition of numeral 5 here.*

This was meant to be a reference to Figure 6 and has been corrected.

*l328. A possibly tidier way to finish this sentence is something like "... a more complex model in this vein would both be difficult to parameterise confidently and be unlikely to fit data meaningfully better than existing models"?*

We have reworded along the lines suggested: “ a more complex model in this vein would be difficult to parameterise confidently while also being unlikely to offer a meaningfully better fit to data than existing models.”

*l331. I think that "who" should be "which", as you're referring to the analysis here, not the people.*

We agree and have corrected accordingly.

*l335. I wonder if "... transmission chain data but, in the absence of this level of detail in the data, branching process models ..." might be a little clearer?*

We have revised with the suggested wording.

*l343. I wonder if "or" or "and/or" would be better than "and" at the start of this line?*

We agree and have changed “and” to “and/or”.

*l359&361. I think "fitted" is preferable to / clearer than "fit" on both these lines.*

We have changed both instances of “fit” to “fitted”.

*l366. Suggest deleting "would like to" - if you'd like to thank them then go ahead and do it :)*

Good suggestion – we have deleted this qualification!

***SuppMat***

*generally (here and in the main manuscript): check and be consistent, you seem to use the term"appendix/appendices" where the journal uses "supplementary material".*

The journal guidelines appear to allow for the term “appendix” (<https://journals.plos.org/ploscompbiol/s/supporting-information>), however we note that the correct usage appears to be “S1 Appendix” rather than “Appendix S1” and we have accordingly corrected all uses of “Appendix S1” to “S1 Appendix”.

*l2 Suggest this paragraph before the section heading "1 Model description" - it's clearly not part of that section!*

We have moved this paragraph out of the “1 Model description” section so that it forms an introductory paragraph for the entire supplement, and have slightly revised the wording to reflect this.

*l11 "During their infectious period, ... total number of contacts ... follows a Poisson distribution". Some more care/detail is required here: depending on what the infectious period distribution is the total number of contacts is generally mixed-Poisson. I think that you mean to say that your model assumes that during the infectious period a Poisson number of contacts are made with mean N.*

This is a good point – we implicitly assumed in writing this description that we were dealing with a fixed infectious period so that the Poisson contact process generated a Poisson-distributed number of contacts, but of course it is possible to still end up with a Poisson-distributed (or approximately Poisson-distributed) number of contacts without the restrictive assumption of a fixed infectious period. We have reworded as follows:

“We assume that during their infectious period an infectious individual makes a Poisson-distributed number of contacts with mean *N*.”

*l32 could write the mean in terms of the new parameters too?*

We have reworded to emphasise that the reformulated model parameter lambda is precisely the distribution mean, and explicitly stated here how the old model parameters alpha_1 and alpha_2 can be expressed in terms of the lambda, Phi, and N.

*l63 the inequality is maybe more helpfully written as N - \lambda + 1 \geq \epsilon \geq 1 ?*

We have rewritten the inequality as $N-\lambda\geq\epsilon-1$ and $\epsilon\geq 1$. We hope that this strikes a balance between emphasising how the inequality follows from Equation (7) while also leading naturally into the interpretation of \epsilon\geq 1 in the following sentence.

*l92 Do you mean "mixed Poisson process" or mixed Poisson *distribution*?*

We did indeed mean Poisson distribution here and have corrected accordingly.

*l122 Suggest saying "probability generating function" here, not just "generating function", since there are many kinds of generating function. Maybe abbreviate to PGF at first use and use the abbreviation afterwards?*

We have specified “probability generating function” in the section heading and at first use, and used the suggested abbreviation.

*l169 I think a little more care is needed in formulating this sentence. The formula (35) gives the limiting variance of the scaled major outbreak size, so the standard deviation you describe in words should have population-size dependence so it can tend to zero.*

This is a good observation. Our original argument was incorrect since the result we were referring to from the textbook by Andersson and Britton concerns the distribution of the attack rate scaled by square root of population size (and also shifted to centre on zero). We have corrected to quote the original result and added the appropriate population size dependence. Our notation ends up being slightly different to that of Andersson and Britton since they take more care to distinguish between the outbreak size inclusive and exclusive of initial cases; in our context there is no need to distinguish between these quantities and so we have avoided introducing extra notation to make this distinction.

*l181 suggest wording "approximate unconditional probabilities of attaining these sizes".*

We have adopted the suggested wording.

*l213 suggest consistency of words/numerals for "zero and 1".*

We have revised the wording to “…between 0 and 1…”.
